# Surgical management of ulnar artery aneurysm in hypothenar hammer syndrome

**DOI:** 10.1590/1677-5449.202400162

**Published:** 2024-12-16

**Authors:** Jonas Almeida dos Santos, Florice de Matos Themótheo, Paulo Henrique Silva Nunes, Marcio Wilker Soares Campelo

**Affiliations:** 1 Universidade Federal do Ceará – UFC, Hospital Universitário Walter Cantídio – HUWC, Fortaleza, CE, Brasil.; 2 Universidade Federal do Ceará – UFC, Fortaleza, CE, Brasil.

**Keywords:** aneurysm, ulnar artery, vascular surgical procedures, surgical anastomosis

## Abstract

Ulnar artery aneurysms are extremely rare and are mainly associated with hypothenar hammer syndrome, an ischemic disorder of the hand resulting from mechanical and repetitive trauma to the hypothenar region. The ulnar artery is hit against the hook of the hamate bone, causing damage to the vessel wall and leading to occlusion or formation of an aneurysm. We describe the case of a truck driver who underwent resection of an ulnar artery aneurysm in the right hand and reconstruction using end-to-end anastomosis with no complications or recurrence.

## INTRODUCTION

Hypothenar hammer syndrome (HHS) is a rare ischemic disorder of the hand caused by thrombosis or aneurysm of the distal ulnar artery and often accompanied by distal digital embolization.^1,2^ Classically, it is associated with repeated mechanical trauma to the hypothenar region.^1^ It primarily affects workers or athletes who perform repetitive movements using the palm of the hand to hit or push. It occurs when the ulnar artery is hit against the hook of the hamate bone, injuring the vessel wall and causing occlusion or aneurysm formation.^3^ Single episodes of acute blunt trauma can also injure the medial wall of the artery, causing HHS.^4^ Incidence is primarily among males, at a proportion of 9:1, at a mean age of 40 years, and the dominant hand is generally involved, in 53-93% of cases.^5,6^

The most common symptoms include pain, paresthesia, weakness, pallor, and susceptibility to cold. More rarely, there can be digital ulceration, necrosis, and gangrene. However, some patients can be asymptomatic.^7^ The severity of symptoms depends on the extent of vascular occlusion and presence of sufficient collateral circulation.^8^ When aneurysm is present, patients may present with a palpable pulsating mass in the area of Guyon’s canal.^5^

This article was approved by the Research Ethics Committee, under consolidated opinion N° 6.486.194 and Ethics Appraisal Submission Certificate: 75373123.5.0000.5045.

## PART I – CLINICAL SITUATION

The patient was a 42-year-old, previously healthy male who described onset approximately 7 months previously of persistent and progressive pain in the right palmar area that progressed to paresthesia, palmar swelling, and cyanosis of the last three fingers. He was right-handed and had been working as a truck driver for almost 20 years. He denied any blunt trauma to the area affected, was a non-smoker, and had no history of vasculitis or other diseases.

**Figure 1:**
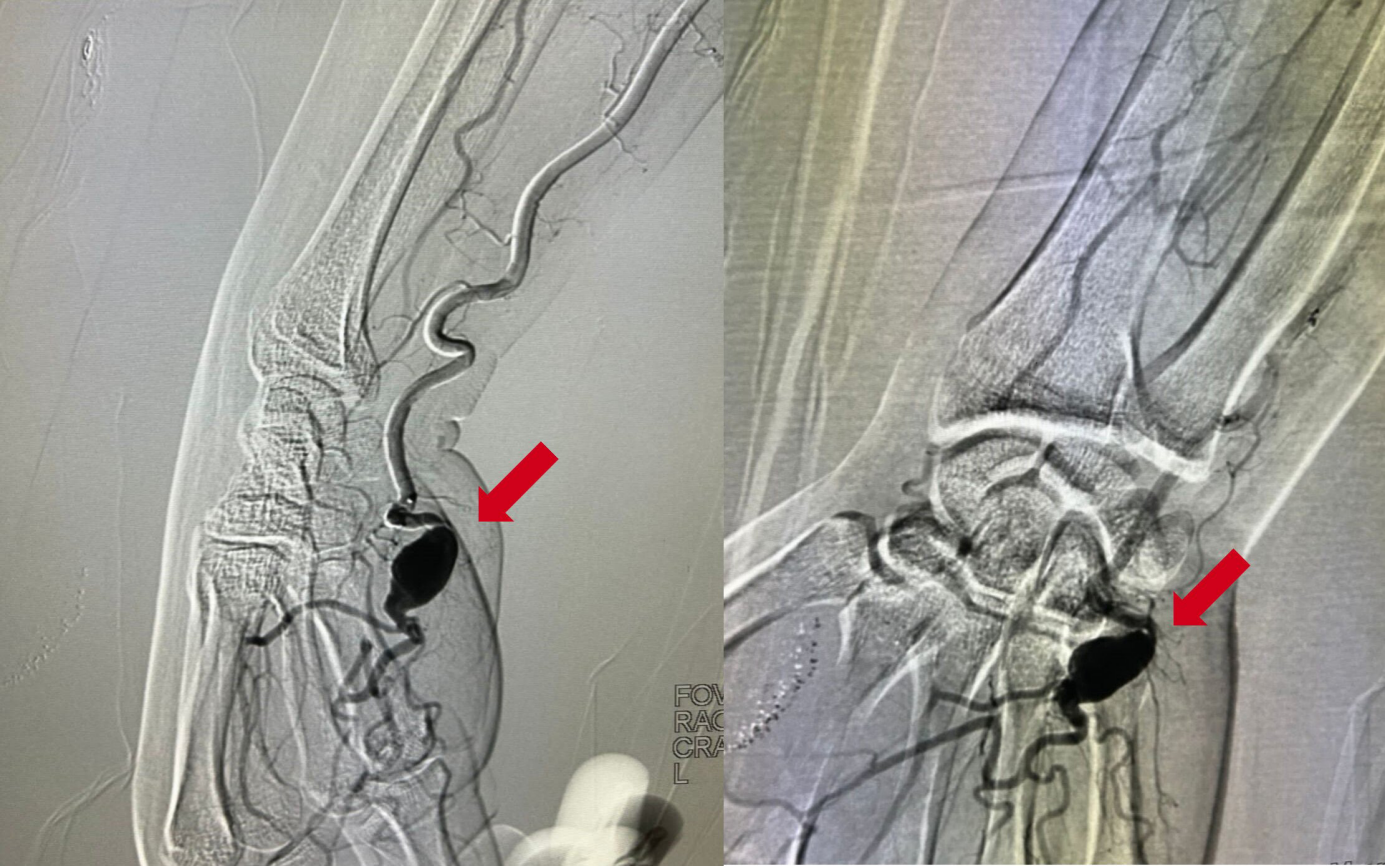
Arteriography image showing the superficial location of the aneurysm in the palm of the right hand.

On examination, a pulsating mass approximately 2 cm in size was observed in the hypothenar area of the right hand, skin was cold, and peripheral cyanosis involving the third, fourth, and fifth fingers. He had no ischemic ulcers on the fingers. Doppler ultrasonography was performed, identifying dilatation of the ulnar artery, compressing Guyon’s canal and measuring 2.6 x 1.5 x 1.2 cm. Next, arteriography (Figure 1) and angiotomography showed a fusiform, partially thrombosed aneurysm of the distal third of the ulnar artery, superficial to the hook of the hamate bone, measuring 1.8 x 1.3 cm and extending approximately 2.6 cm. The distal third of the brachial artery and its other branches, including the radial artery, were normal in both course and caliber. Bony structures and soft tissues were free from changes.

## PART II – WHAT WAS DONE

**Figure 2:**
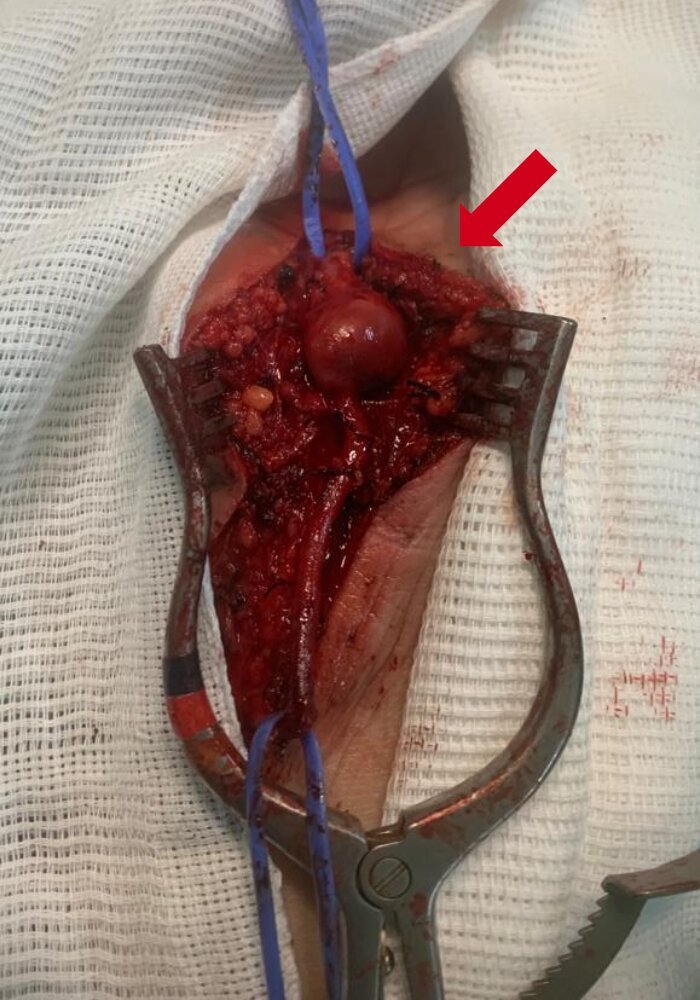
Intraoperative images showing the position of the ulnar artery and the aneurysm before resection.

**Figure 3:**
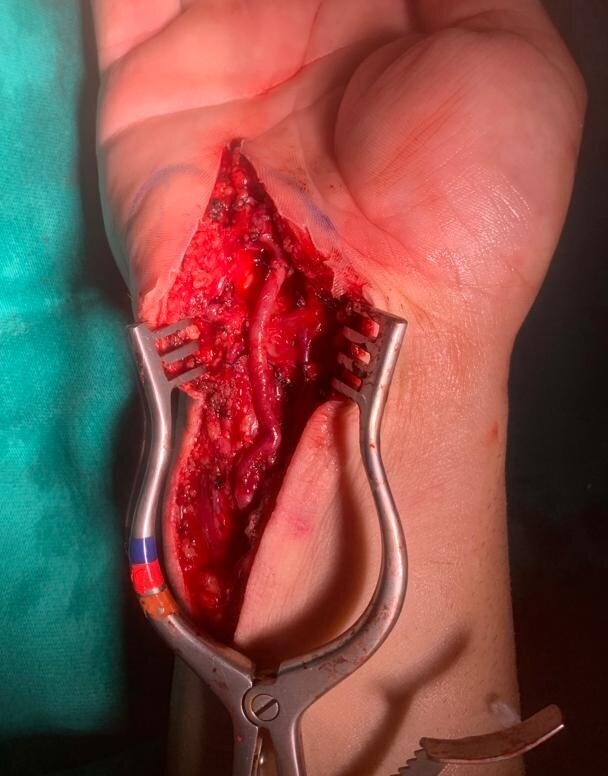
Appearance of the ulnar artery after resection and reconstruction with end-to-end anastomosis.

Surgical access was obtained via a longitudinal incision along the path of the ulnar artery, extended lateral to the aneurysm. The surgical field was explored layer by layer and the ulnar artery was identified and then isolated proximally and distally (Figure 2). Aneurysmectomy was performed, followed by reconstruction with end-to-end anastomosis of the artery (Figure 3). The tortuosity of the ulnar artery in the distal forearm enabled it to be tractioned sufficiently to compensate for the length of the segment lost when resecting the aneurysm. The length of the incision in the forearm and the extent of dissection of the ulnar artery were sufficient for it to be displaced in this manner. There was no need for ligation of other branches of the displaced segment, enabling the artery to be tractioned.

**Figure 4:**
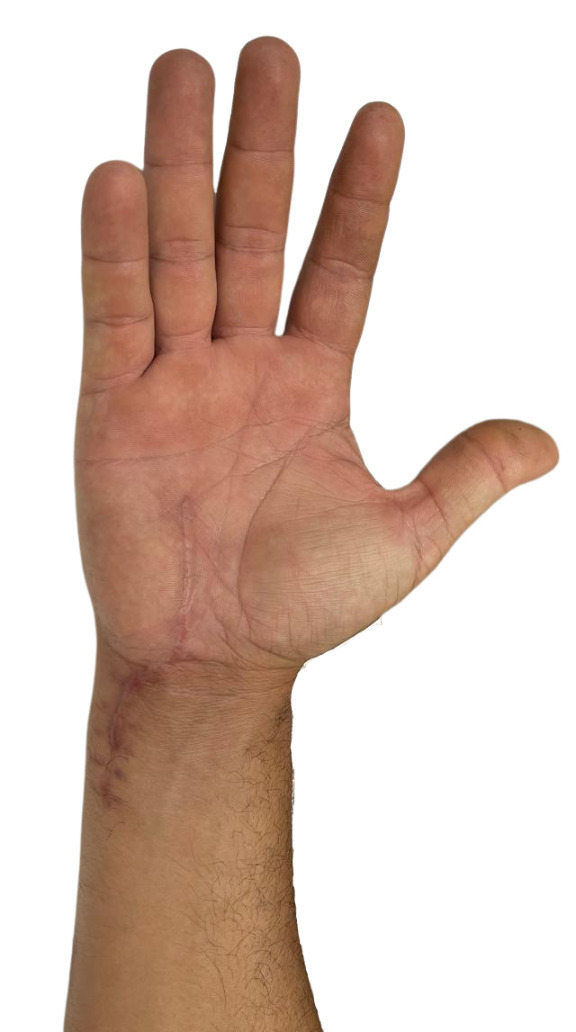
Image of the patient’s hand 10 months after surgery, without recurrence of pain or of the aneurysm.

During the immediate postoperative period, the patient’s hand movements were mildly restricted in order to facilitate the healing process. During the late postoperative period (Figure 4), the patient was stable, with no impaired mobility or postoperative complications. The patient remains in six-monthly outpatient follow-up at our service.

## DISCUSSION

It is believed that HHS is the result of a combination of the anatomic characteristics of the ulnar artery and repetitive traumas to the palmar region.^5^ The artery’s superficial course through this region makes it relatively susceptible to mechanical injuries.^9^ Blows and traumas to the palmar region are relatively common in the context of work, but HHS and ulnar artery aneurysm are rare. Some authors have thus raised the hypothesis that a preexisting intrinsic abnormality of the ulnar artery is an additional risk factor. Possible abnormalities include fibromuscular dysplasia of the wall of the vessel, intimal hyperplasia, ectasia, and anatomic variants.^2,7,9^ Other causes of aneurysms in the extremities include infections, vasculitis, connective tissue diseases, iatrogenic injury, or idiopathic cases.^10^

It is recommended that an occupational history is taken during diagnostic assessment, to raise the degree of suspicion. When they occur, ulnar artery aneurysms can present as pulsating masses in the middle of the palm of the hand. Raynaud’s phenomenon may also be present.^3^ However, many patients with ulnar artery aneurysm do not have a palpable mass and the Allen test may be normal if the aneurysm is not occlusive.^5^ Angiotomography is a widely used method that enables simultaneous investigation of both hands, enabling comparison of the vascular architecture, in addition to showing details of bony structures and soft tissues.^4^ However, angiography is still considered the gold-standard method to determine the nature, degree, and location of aneurysms of the extremities.^2,4^ Additionally, the procedure can reveal the presence of digital emboli and rule out other differential diagnoses.^6^ Doppler ultrasonography can be used to evaluate arterial hemodynamics, to determine the site of origin of thrombus,^8^ and to differentiate pseudoaneurysms from hematoma.^3^ Another advantage of this method is that it does not induce vasospasm.^8^ In atypical HHS presentations, laboratory tests can be considered as a means to rule out autoimmune or hematological causes.^1^ Authors such as Yuen et al. have proposed algorithms to support diagnosis of HHS, based on a wide-ranging review of the literature available on the subject.^11^

The approach to treatment of HHS is still controversial because of the rarity of the disease, which limits the scope for systematic studies comparing different techniques, and also because of the wide spectrum of the symptomology and severity of disease presentation.^2,8,11^ Intra-arterial thrombolysis can achieve good results in patients with acute or subacute cases (days or months). Thrombolytic agents such as recombinant tissue plasminogen can be administered via selective catheterization, eliminating the thrombosis and its symptoms. Thrombolysis can also be administered as a preoperative therapy with the objective of reducing the quantity of thrombi prior to surgery.^1,2,4^ Conservative treatment may be indicated for patients who are not at immediate risk of tissue loss, necrosis, or active ischemia. This approach can also be employed as an adjuvant to surgical treatment.^4^ Options include lifestyle changes, such as smoking cessation. Calcium channel blockers, beta blockers, and alpha-blockers, which act as vasodilators, can be used to reduce the sympathetic tone, vasospasms, and Raynaud’s phenomenon, and antiplatelet drugs, anticoagulants, and padded protective gloves can also be used.^1,2,4,8^ If the condition is not treatable with conservative management, such as when an aneurysm is present, then surgical treatment will be needed.^6^

In general, the objective of surgical treatment is to restore blood flow to the ischemic fingers and prevent thrombotic or embolic complications.^4^ Surgical options include ligation of the aneurysm, resection with end-to-end primary anastomosis, or reconstruction with a venous or arterial graft.^12^ The choice of technique depends on clinical assessment of the patient, the location of the aneurysm, the symptoms, and the degree of concern with formation of thrombi, embolization, and the possibility of arterial occlusion.^10,12^ It is important to determine the size of the aneurysm to plan vascular reconstruction and it is recommended that even when asymptomatic aneurysms should be resected to avoid embolization.^13^ The abundant collateral circulation in the hand means that ligation can be tolerated in cases in which reconstruction cannot be performed.^3^ For distal aneurysms, simple resection with ligation of the artery is possible if the hand is adequately perfused via an intact radial artery. However, if perfusion of the hand is inadequate, reconstruction of the ulnar artery is obligatory. When the vessel is not under tension, reconstruction can be by primary end-to-end anastomosis.^10^ When needed, interposition grafts can be obtained by harvesting a small vein with narrow caliber, such as the saphenous, cephalic, or basilic.^3^ However, arterial grafts and direct arterial repairs achieve better rates of patency.^5^

Hypothenar hammer syndrome may be associated with development of an ulnar artery aneurysm in the hypothenar region. Diagnosis is by imaging exams such as angiography and Doppler combined with an occupational history of repeated blunt trauma to the affected hand. In selected patients, ulnar artery aneurysms can be treated surgically with resection of the aneurysm and reconstruction by end-to-end anastomosis with satisfactory results.

## References

[1] Jud P, Pregartner G, Berghold A (2021). Endovascular thrombolysis in hypothenar hammer syndrome: a systematic review. Front Cardiovasc Med.

[2] Seldén A, Hermiz F, Östlund B. (2016). Hammarsjuka är ovanligt – eller bara ett ovanligt förbisett tillstånd - Effektiv behandling finns – skärpt differentialdiagnostik är motiverad. Lakartidningen.

[3] Buda SJ, Johanning JM (2005). Brachial, radial, and ulnar arteries in the endovascular era: choice of intervention. Semin Vasc Surg.

[4] Hui-Chou HG, McClinton MA (2015). Current options for treatment of hypothenar hammer syndrome. Hand Clin.

[5] McClinton MA (2011). Reconstruction for ulnar artery aneurysm at the wrist. J Hand Surg Am.

[6] Ravari H, Johari HG, Rajabnejad A (2018). Hypothenar hammer syndrome: surgical approach in patients presenting with ulnar artery aneurysm. Ann Vasc Surg.

[7] Gala ZS, Ayyala H, Viviano SL, Ignatiuk A (2020). Saved by the SPY: ulnar artery reconstruction with LCFA graft for hypothenar hammer syndrome. Eplasty.

[8] Estíbaliz AT, Eztizen LS, Osman-Alberto SA (2022). Bilateral hypothenar hammer syndrome case presentation and literature review. Case Rep Vasc Med.

[9] Ferris BL, Taylor LM, Oyama K (2000). Hypothenar hammer syndrome: proposed etiology. J Vasc Surg.

[10] Abdulla YKA, Kamal DM, Mathew KP (2021). Idiopathic true ulnar artery aneurysm. Int J Surg Case Rep.

[11] Yuen JC, Wright E, Johnson LA, Culp WC (2011). Hypothenar hammer syndrome: an update with algorithms for diagnosis and treatment. Ann Plast Surg.

[12] Hart J, Hajjar R, Laveroni E (2021). Hypothenar hammer syndrome and repair of ulnar artery aneurysm in a patient without history of trauma. BMJ Case Rep.

[13] Titan AL, Chang J, Megerle K, Murray P, Hammert W (2023). State of the art review: the management of chronic vascular disorders in the hand and upper limb. J Hand Surg Eur Vol.

