# Supplementary material for: Surgical management of ulnar artery aneurysm in hypothenar hammer syndrome
Source: J Vasc Bras. 2024 Dec 16;23:e20240016. doi: 10.1590/1677-5449.202400162 (PMC11702975; doi:10.1590/1677-5449.202400162)
Supplement: Translation

# Translation: Abordagem cirúrgica de aneurisma de artéria ulnar na síndrome do martelo hipotenar

**DOI:** 10.1590/1677-5449.202400161

**Authors:** Jonas Almeida dos Santos, Florice de Matos Themótheo, Paulo Henrique Silva Nunes, Marcio Wilker Soares Campelo

**Affiliations:** 1 Universidade Federal do Ceará – UFC, Hospital Universitário Walter Cantídio – HUWC, Fortaleza, CE, Brasil.; 2 Universidade Federal do Ceará – UFC, Fortaleza, CE, Brasil.

**Keywords:** aneurisma, artéria ulnar, procedimentos cirúrgicos vasculares, anastomose cirúrgica

## Abstract

Os aneurismas de artéria ulnar são extremamente raros e estão associados, principalmente, com a síndrome do martelo hipotenar (SMH), que é um distúrbio isquêmico da mão decorrente de traumas mecânicos e repetitivos na região hipotenar. Ocorre quando a artéria ulnar é atingida contra o gancho do osso hamato, originando lesão da parede do vaso, ocasionando oclusão ou formação de aneurisma. Descrevemos o caso de um paciente, motorista de caminhão, submetido a ressecção de aneurisma de artéria ulnar da mão direita com reconstrução por anastomose término-terminal sem intercorrências ou recidiva.

## INTRODUÇÃO

A síndrome do martelo hipotenar (SMH) é um raro distúrbio isquêmico da mão causado por trombose ou aneurisma na artéria ulnar distal, frequentemente acompanhado de embolização digital distal^1,2^ . Classicamente, está associada a trauma mecânico repetitivo na região hipotenar^1^ . Ocorre principalmente em trabalhadores ou atletas que realizam movimentos repetitivos de bater ou empurrar com a palma da mão. Surge quando a artéria ulnar é atingida contra o gancho do osso hamato, originando lesão da parede do vaso, ocasionando oclusão ou formação de aneurisma^3^ . Episódios agudos únicos de trauma contuso também podem danificar a parede medial da artéria, causando a SMH^4^ . Ocorre principalmente no sexo masculino, em uma proporção de 9:1, com idade média de 40 anos, e envolve comumente a mão dominante, com ocorrência em 53-93% dos casos^5,6^ .

Os sintomas mais comuns incluem: dor, parestesia, fraqueza, palidez e intolerância ao frio. Mais raramente, inclui ulceração, necrose e gangrena digital. Entretanto, alguns pacientes podem ser assintomáticos^7^ . A gravidade dos sintomas depende da extensão do acometimento vascular ocluído e da presença de circulação colateral suficiente^8^ . Quando há aneurisma, os pacientes podem apresentar massa pulsátil palpável na área do canal de Guyon^5^ .

O presente artigo foi aprovado pelo Comitê de Ética em Pesquisa, sob o parecer consubstanciado N° 6.486.194, Certificado de Apresentação de Apreciação Ética: 75373123.5.0000.5045.

## PARTE I – SITUAÇÃO CLÍNICA

O paciente é do sexo masculino, de 42 anos de idade, previamente hígido, e relata que há aproximadamente 7 meses teve princípio de quadro de dor persistente e progressiva na região palmar direita, que evoluiu com parestesia, abaulamento palmar e cianose dos três últimos dedos da mão. Relata ser destro e motorista de caminhão há quase 20 anos. Também, nega trauma contuso na região afetada, não é tabagista e não possui antecedentes de vasculites ou outras doenças.

Ao exame, percebe-se massa pulsátil de aproximadamente 2 cm na região hipotenar da mão direita, pele fria e cianose não fixa no terceiro, quarto e quinto quirodáctilo. Não apresentava úlceras isquêmicas nos dedos. Foi solicitada ultrassonografia com Doppler que identificou dilatação da artéria ulnar, comprimindo o canal de Guyon, medindo 2,6 x 1,5 x 1,2 cm. Em seguida, arteriografia (Figura 1) e angiotomografia mostraram aneurisma fusiforme parcialmente trombosado no terço distal da artéria ulnar, localizado superficialmente ao hâmulo do osso hamato, medindo 1,8 x 1,3 cm, com extensão de cerca de 2,6 cm. Terço distal da artéria braquial e seus demais ramos, incluindo a artéria radial, exibem trajeto e calibre normais. Estruturas ósseas e partes moles não apresentam alterações.

**Figura 1:** Imagem de arteriografia mostrando a localização superficial do aneurisma na região palmar da mão direita.

## PARTE II – O QUE FOI FEITO

A abordagem cirúrgica foi realizada por meio de incisão longitudinal no trajeto da artéria ulnar, se continuando lateral ao aneurisma. Foi realizada a exploração por planos e identificada a artéria ulnar, na qual foi isolada proximal e distalmente (Figura 2). Efetuou-se a aneurismectomia, seguida da reconstrução por anastomose término-terminal da artéria (Figura 3). A tortuosidade da artéria ulnar na porção distal do antebraço permitiu a tração da mesma para suprir o comprimento sacrificado do segmento ressecado do aneurisma. A incisão no antebraço e a dissecção da artéria ulnar ocorreu por uma extensão longa para permitir a sua mobilização. Não houve necessidade de ligadura de outros ramos nesse segmento mobilizado, permitindo a tração da artéria.

**Figura 2:** Imagens do intraoperatório mostrando a delimitação da artéria ulnar e o aneurisma antes da ressecção.

**Figura 3:** Aspecto da artéria ulnar após a ressecção e reconstrução com anastomose término-terminal.

No pós-operatório imediato, foi realizada uma leve restrição dos movimentos da mão do paciente para favorecer o processo de cicatrização. No pós-operatório tardio (Figura 4), o paciente evoluiu estável, sem alterações de mobilidade ou complicações pós-operatórias. O paciente permanece em seguimento ambulatorial semestral em nosso serviço.

**Figura 4:** Imagem da mão do paciente 10 meses após a cirurgia, sem recorrência de dor ou aneurisma.

## DISCUSSÃO

Acredita-se que a SMH seja decorrente da combinação entre as características anatômicas da artéria ulnar com traumas repetitivos na região palmar^5^ . O trajeto superficial da artéria nessa região torna o vaso relativamente propenso a lesões mecânicas^9^ . No contexto ocupacional, os golpes e traumas na região palmar são relativamente comuns, entretanto, a SMH e o aneurisma de artéria ulnar são raros. Desse modo, alguns autores levantaram a hipótese de anormalidade intrínseca preexistente da artéria ulnar como fator de risco adicional. Entre essas anormalidades estão a displasia fibromuscular na parede do vaso, hiperplasia intimal, ectasias ou variantes anatômicas^2,7,9^ . Outras causas de aneurismas de extremidades incluem infecções, vasculites, doenças do tecido conjuntivo, iatrogenias ou idiopáticas^10^ .

Recomenda-se incluir a história ocupacional durante a avaliação diagnóstica para aumentar o grau de suspeição. Quando presentes, os aneurismas de artéria ulnar podem apresentar-se como uma massa pulsátil no meio da palma da mão. O fenômeno de Raynaud também pode estar presente^3^ . Entretanto, muitos pacientes com aneurisma de artéria ulnar não apresentam massa palpável e o teste de Allen pode ser normal se o aneurisma for não oclusivo^5^ . A angiotomografia é um método amplamente utilizado que permite a avaliação simultânea de ambas as mãos, possibilitando a comparação da arquitetura vascular, além de oferecer detalhes de estruturas ósseas e partes moles^4^ . Entretanto, a angiografia ainda é considerada o método padrão-ouro para determinar a natureza, grau e localização dos aneurismas de extremidades^2,4^ . Além disso, o procedimento pode evidenciar a existência de êmbolos digitais e descartar outros diagnósticos diferenciais^6^ . A ultrassonografia Doppler possibilita avaliar a hemodinâmica arterial, determinar o local de origem do trombo^8^ e diferenciar falsos aneurismas de hematomas^3^ . Outra vantagem desse método é que não induz vasoespasmos^8^ . Em apresentações atípicas da SMH, exames laboratoriais podem ser considerados para descartar causas autoimunes ou hematológicas^1^ . Autores como Yuen et al. propuseram algoritmos para auxiliar no diagnóstico da SMH, baseando-se em uma ampla revisão da literatura disponível sobre o assunto^11^ .

A abordagem terapêutica da SMH ainda é controversa devido à raridade da doença, o que limita a realização de estudos sistemáticos comparando as diferentes técnicas, além do amplo espectro de sintomatologia e gravidade de apresentações da doença^2,8,11^ . A trombólise intra-arterial pode ser usada em pacientes com quadro agudo ou subagudo (dias ou meses) com bons resultados. Agentes trombolíticos como plasminogênio tecidual recombinante podem ser administrados via cateter seletivamente, eliminando a trombose e os sintomas. A trombólise também pode ser administrada como terapia pré-operatória com o objetivo de diminuir a quantidade de trombos antes da cirurgia^1,2,4^ . O tratamento conservador pode ser indicado para pacientes que não apresentam risco iminente de perda tecidual, necrose ou isquemia ativa. Essa abordagem também pode ser usada de maneira complementar ao tratamento cirúrgica^4^ . As opções incluem modificação de estilo de vida, como cessação do tabagismo. Bloqueadores de canais de cálcio, betabloqueadores e alfa-bloqueadores, que atuam como vasodilatadores, podem ser utilizados para diminuir o tônus simpático, vasoespasmos e o fenômeno de Raynaud, assim como antiplaquetários, anticoagulantes e luvas de proteção acolchoadas^1,2,4,8^ . Se a condição não puder ser tratada de forma conservadora, como na presença de aneurisma, o tratamento cirúrgico é indicado^6^ .

De maneira geral, o tratamento cirúrgico tem o objetivo de restaurar o fluxo sanguíneo para os dedos isquêmicos e prevenir complicações trombóticas ou embólicas^4^ . As opções cirúrgicas incluem a ligadura do aneurisma, ressecção com anastomose término-terminal primária ou reconstrução com enxerto de veia ou artéria^12^ . A escolha da técnica depende da avaliação clínica do paciente, da localização do aneurisma, dos sintomas e da preocupação com formação de trombos, embolização e possível oclusão arterial^10,12^ . É importante determinar a extensão do aneurisma para planejar a reconstrução vascular e, mesmo assintomáticos, recomenda-se que os aneurismas sejam ressecados para evitar embolização^13^ . Graças à abundante circulação colateral na mão, a ligadura pode ser tolerada em casos no qual a reconstrução não pode ser realizada^3^ . Em aneurismas distais, a ressecção simples com ligadura da artéria é possível se a mão estiver adequadamente perfundida por uma artéria radial intacta. Entretanto, se a perfusão da mão for inapropriada, a reconstrução da artéria ulnar é obrigatória. Quando não houver tensão, a reconstrução pode ser realizada por anastomose término-terminal primária^10^ . Quando necessários**,** os enxertos de interposição podem ser obtidos pela coleta de uma pequena veia de pequeno calibre, como a safena, cefálica ou basílica^3^ . Entretanto, enxertos arteriais e reparo arterial direto apresentaram maior taxa de patência^5^ .

A SMH pode estar associada ao surgimento de aneurisma da artéria ulnar na região hipotenar. Seu diagnóstico depende de exames de imagem como angiografia e Doppler aliados a uma história ocupacional de trauma contuso e repetitivo na mão afetada. Em pacientes selecionados, os aneurismas de artéria ulnar podem ser tratados cirurgicamente com ressecção do aneurisma e reconstrução por anastomose término-terminal com resultados satisfatórios.
